# Is Age of 80 Years a Threshold for Carotid Revascularization?

**DOI:** 10.2174/157340311795677716

**Published:** 2011-02

**Authors:** Boudewijn L Reichmann, Guus W. van Lammeren, Frans L Moll, Gert J. de Borst

**Affiliations:** Department of Vascular Surgery, University Medical Centre Utrecht; The Netherlands

**Keywords:** Carotid revascularization, angioplasty, stenting, octogenarians, demographic features, revascularization.

## Abstract

**Background and purpose:**

Carotid Angioplasty and Stenting (CAS) has emerged as an alternative to Carotid Endarterectomy (CEA) in treatment of carotid stenotic disease. With increasing life expectancy clinicians are more often confronted with patients of higher age. Octogenarians were often excluded from randomized trials comparing CAS to CEA because they were considered high-risk for revascularization. Conflicting results on the peri-procedural outcome of carotid revascularization in these patients have been reported. In order to objectively evaluate whether age above 80 years should be an upper limit for indicating carotid revascularization we systematically reviewed the currently available literature.

**Methods:**

Literature was systematically reviewed between January 2000 and June 2010 using Pubmed and Embase, to identify all relevant studies concerning CAS and CEA in octogenarians. Inclusion criteria were 1) reporting outcome on either CEA or CAS; and 2) data subanalysis on treatment outcome by age. The 30-day Major Adverse Event (MAE) rate (disabling stroke, myocardial infarction or death) was extracted as well as demographic features of included patients.

**Results:**

After exclusion of 23 articles, 46 studies were included in this review, 18 involving CAS and 28 involving CEA. A total of 2.963 CAS patients and 14.365 CEA patients with an age >80 years were reviewed. The MAE rate was 6.9% (range 1.6 - 24.0%) following CAS and 4.2% (range 0 – 8.8%) following CEA.

A separate analysis in this review included the results of one major registry 140.376 patients) analyzing CEA in octogenarians only reporting on 30-day mortality and not on neurological or cardiac adverse events. When these data were included the MAE following CEA is 2.4% (range 0 – 8.8%)

**Conclusions:**

MAE rates after CEA in octogenarians are comparable with the results of large randomized trials in younger patients. Higher complication rates are described for CAS in octogenarians. In general, age > 80 years is not an absolute cut off point to exclude patients from carotid surgery. In our opinion, CEA should remain the golden standard in the treatment of significant carotid artery stenoses, even in the very elderly.

## INTRODUCTION

Carotid Angioplasty and Stenting (CAS) has emerged as an alternative to Carotid Endarterectomy (CEA) for the treatment of carotid artery stenoses in the prevention of stroke ^1^. Recent results of large randomized trials have shown that CAS however has a higher peri-procedural complication rate compared to CEA [2-4]. The authors concluded that CAS should only be considered in high-risk patients not suitable for surgery and that CEA remains the gold standard until long-term results of randomized trials can be reported. 

The question remains which patients should be considered as high-risk patients. Previous neck surgery, prior neck cancer with radiation therapy, clinical significant cardiopulmonary disease or an age above 80 have been exclusion criteria in many large carotid trials that were conducted to evaluate the durability of CEA for the prevention of stroke [5-9]. Age has also been identified as an independent predictor of complications in carotid interventions [10-12]. Mean life expectancy has steadily increased over time, and elderly people have become the fastest growing population segment in industrialized countries. Hence, elderly people are typically seen in everyday clinical practice and will become an increasingly important group of patients in the future. “High-risk” patients were often offered the endovascular alternative to surgical treatment because CAS is considered to be a less invasive revascularization option. Some authors state that CAS can be performed safely in high-risk patients [13-15], but subgroups often are too small to draw any conclusions on the procedural risk of for example patients of higher age. Octogenarians however, were excluded from most trials evaluating CAS. To date, there has not been a randomized trial comparing CAS with CEA in octogenarians. 

In order to answer two specific questions; 1) is carotid revascularization in octogenarians safe to perform; and 2) is CAS compared to CEA a safer treatment option for octogenarians in terms of perioperative MAE; we systematically reviewed current literature on carotid revascularization in octogenarians. 

## METHODS

Literature was searched to identify all relevant studies on carotid revascularization in octogenarians. The search was restricted to papers published between January 2000 and June 2010. Studies were initially identified from the Medline/Pubmed database, EMBASE and the Cochrane database using the search terms “carotid stenosis”, “carotid angioplasty”, “carotid stenting”, “carotid revascularization”, “carotid endarterectomy”, “octogenarians” and “(very) elderly”.

Studies were included if they reported on octogenarians treated by CAS or CEA. Results of major adverse events (MAE) (disabling stroke, myocardial infarction or death) had to be described in order to be included. Studies however were excluded if: 1) The age of the patient population was <80 years, 2) review articles or letters did not describe rates of major adverse events, 3) articles were written in non-English language . The reference list of the included articles was also screened for additional studies concerning our subject. Data on 30-day MAE rates as well demographic features of the included patients were extracted from the articles and analyzed. 

## RESULTS

### Search Results

**Fig. (1):**
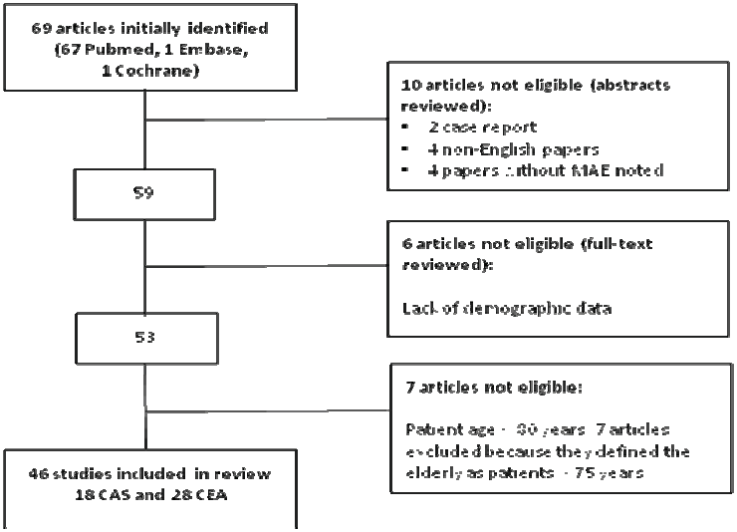
Flow chart of search results.

Sixty-nine studies were initially identified with our search strategy (Fig. **1**; flow chart). Of these 69 initial articles, 10 articles did not meet our inclusion criteria, 2 were case reports, 4 articles were written in non-English languages and 4 articles lacked description of Major Adverse Event (MAE). Six articles were excluded because there was a lack of demographic data on included patients and 7 articles described carotid revascularization in a patient population with an average age <80 years. After exclusion 46 articles met our inclusion criteria (18 CAS and 28 CEA) [16-60]. 

### Carotid Angioplasty and Stenting in Octogenarians

The combined amount of CAS procedures performed in the included articles was 10.896. Of these procedures 2.837 were performed in octogenarians. Demographic features and an outline of included articles are shown in Table **1**. Forty-nine percent of patients treated by CAS were symptomatic. In two series, a significant higher complication rate was observed in symptomatic octogenarians compared to asymptomatic octogenarians [21, 24]. 

Other series included in this review did not analyze the difference between symptomatic and asymptomatic octogenarians. An Embolic Protection Device (EPD) was used in 86% of the patients. Usman *et al* who conducted a meta-analysis on carotid revascularization in octogenarians describe a review of 826 patients ^19^. They concluded that octogenarians undergoing CAS have a 3.46-times higher absolute risk of stroke than those undergoing CEA, with no significant difference in mortality and a trend toward a lower rate of myocardial infarction. The rate of MAE (disabling stroke, myocardial infarction or death) varied from 1,6 to 24%. The total number of MAE was 206 which corresponds to a combined MAE rate of 6,9% in the 2.837 included patients. These MAE’s were mainly stroke related; myocardial infarction was relatively rare following CAS. Zahn *et al* reported only non-fatal strokes and death and did not analyze cardiac complications ^24^. Their MAE rate of 5,5% is therefore probably underestimated. 

An age > 80 years is as an independent risk factor for peri-procedural MAE [25, 27, 31], and thus age over 80 was an exclusion criterium in many carotid trials. This policy however was not endorsed by the results of other authors in this review. They did not observe significant increases in complication rates of octogenarians compared to younger patients. 

The use of an EPD is essential in the prevention of peri-procedural cerebrovascular events according to three authors, because of the observed higher event rate compared to unprotected CAS procedures [23, 24, 26]. 

### Carotid Endarterectomy in Octogenarians

Much more data are available on CEA in octogenarians. The included articles and their demographic features are outlined in Table **2**. The by far largest cohort was reported by Lichtman *et al*. who analyzed data of all 140.376 patients older than 80 years undergoing CEA in the United States during a period of 6 years (1993-1999) [33]. They collected data on patients from their medical records. Their group reported a 30-day mortality rate of 2,2% but did not specify the cause of death nor did they report on cerebrovascular complication rates. Ninety-three of the total included patients in this review are coming from their patient population. If this group is included the combined MAE rate would be 2,4%. This however considerably underestimates the exact MAE rates because of the lack of figures about neurological and cardiac complications. 

When the study of Lichtman is not included, a patient population of 60.060 containing 14.365 octogenarians remains for analysis. In total 47% of the included patients undergoing CEA were symptomatic. The MAE rates in the included articles varied between 0-8,3%. The total number of MAE was 606 on a total of 14.365 CEA’s in patients older than 80. The combined MAE rate was 4.2%. Two authors describe that especially symptomatic octogenarians are more at risk for peri-procedural complications compared to asymptomatic patients undergoing CEA [39, 59]. 

## DISCUSSION

With the increasing life expectancy clinicians are more often confronted with elderly patients affected by carotid obstructive disease. It has been estimated that 30-40% of strokes in octogenarians are secondary to stenotic or occlusive disease of the carotid bifurcation [61]. Carotid revascularization in the elderly remains controversial and conflicting results on peri-procedural outcome have been reported. Two studies however, have shown that, on average, 80% of octogenarians survive at least 4 years after endarterectomy and that the vast majority is stroke free at 5 to 10 years follow-up [58, 62]. Norman *et al* conclude that the likelihood of living long enough to gain benefit from a carotid endarterectomy is not jeopardized by being too old [49]. Elderly patients with a symptomatic carotid stenosis treated by best medical treatment have the highest risk on future cerebrovascular events [63]. It might therefore be beneficial to offer any carotid revascularization, whether surgical or endovascular, to octogenarians to decrease this relatively high risk of (recurrent) stroke. 

The result of this systematic review shows that octogenarians have an increased risk of major adverse events during CAS compared to CEA. A recent meta-analysis concerning carotid revascularization in octogenarians showed that the peri-procedural all-stroke rate was significantly higher during CAS [19]. The absolute risk on stroke was 3.46-times higher compared to patients undergoing CEA. There was also a trend towards higher mortality and myocardial infarction rates but these results did not reach statistical significance. Several other authors like the CREST investigators endorse the conclusion of this meta-analysis. After interim analysis of the results of the lead-in phase of the CREST trial the inclusion of octogenarians was stopped. Octogenarians showed a 30-day stroke/death rate of 12.1% compared to 3.2% in younger patients [30]. 

Other authors invalidate inferiority of CAS in octogenarians and showed excellent results in their patient populations [16, 18, 20, 28]. A recent meta-analysis by Bonati *et al*. containing the pooled data of three recent large randomized trials confirmed the significant higher complication rates following CAS in patients > 75 years [64] 

The explanation for this increase in major adverse events after CAS compared to CEA is poorly defined. Anatomic characteristics might play an important role in the occurrence of major adverse events. Octogenarians have an increased incidence of complex anatomic risk factors compared to younger patients [25, 65]. Lam *et al* have described several of these characteristics. They concluded that octogenarians have an increased incidence of unfavorable arch elongation, arch calcification, common carotid or innominate artery origin stenosis, common carotid artery tortuosity, and internal carotid artery tortuosity. Increased arch calcium content and type II aortic arches may be markers of increased potential for embolization during endovascular manipulation that transverses the aortic arch [66]. The rate of embolic events during CAS is considered to decrease when an embolic protection device (EPD) is used but preliminary manipulation of interventional devices through a calcified aortic arch might already have contributed to cerebral lesions, prior to EPD placement. The discussion on the standard use of CPD not closed; also in the light of recent findings that new cerebral infarctions were higher in CAS than in CEA, especially in CPD assisted CAS [67]. Kastrup *et al* also described the correlation between the incidence of new lesions on diffusion–weighted imaging and aortic arch calcification in the elderly [68].

Another explanation might be found in plaque characteristics at the target site. There are no data available in the current literature reporting on specific carotid plaque characteristics in octogenarians, but plaque stability has been reported to decrease with age [69]. It might be conceivable that the underlying plaque composition in octogenarians is more unstable and rupture prone, compared to carotid plaques in younger patients, which might contribute to the increased risk for thrombo-embolic events during CAS, due to plaque disruption initiated by endovascular devices and stents. Our study group is currently conducting a study concerning plaque stability in octogenarians compared to younger patients, but results have to be awaited.

Embolization is not uncommon during CAS. The use of EPD might prevent some events but the embolization can occur during each step of the procedure. A lot of these micro-emboli occur subclinically but some factors could provoke subclinical events and lesions to become clinical. One of these factors is cerebral reserve but data concerning the effect of cerebral reserve on outcome of carotid revascularization is poorly defined in octogenarians. Chaer *et al* studied cerebral reserve and saw that an age >/=70 is associated with poor cerebral reserve in patients with significant carotid stenosis as measured by Cerebral Blood Flow response to an acetazolamide challenge [70]. This poor reserve might make older patients more sensitive to micro-emboli and therefore explain the higher risk of stroke during CAS compared to CEA. 

Interventionalists often attribute higher rates of major adverse events during CAS in large randomized trials to the fact that less experienced interventionalists are compared to experienced surgeons. Experienced interventionalists are more likely to recognize treacherous anatomy and make adjustments to minimize procedural risks than less experienced operators. Patient selection and a well considered choice for an either surgical or endovascular approach remains a key factor in carotid revascularization. Some authors therefore believe that when appropriate patient selection and evaluation of their preoperative risk factors is performed equal peri-procedural results can be achieved in CAS and CEA in high risk patients ^71^ So far, however, the data derived in this review concluded otherwise.

Whether or not a patient is symptomatic could attribute to the risk of major adverse events. A carotid artery stent registry noted a significant difference in stroke rates in symptomatic octogenarians of 7.1% versus 3.9% in younger symptomatic patients. This relevant difference was not found in asymptomatic patients (3.4% vs. 2.6%). Other authors, including the CREST investigators, did not find an increased peri-procedural complication rate between symptomatic and asymptomatic octogenarians [30]

The data obtained in this review show a 30-day MAE rate of 4.2% following CEA in octogenarians. This MAE rates are consistent with complication rates of recent large randomized trials in non-octogenarians [2-4]. It seems a consistent finding that CEA can be safely performed in the very elderly with equal complication rates compared to a younger population. 

This review is limited by the age cut-off point. On purpose, we focused on octogenarians (age > 80 years) whereas some authors use an age > 75 years to define elderly in their studies. Our main focus was on octogenarians and therefore we had to discard and exclude 7 articles from our review.

## CONCLUSION

Age is not a criterion to withhold patients from surgery. CEA in both symptomatic and asymptomatic octogenarians can be performed with comparable and acceptable peri-procedural complication rates as in younger patients. Higher complication rates in patients older than 80 years occur with CAS. Therefore, CEA must remain the gold standard in the treatment of carotid occlusive disease, also in patients above the age of 80.

## Figures and Tables

**Table 1:** Carotid Angioplasty and Stenting (CAS) Study Collection and 30-day MAE Rates.

First author	Year	Total CAS	Octogenarians, N	Male	Mean age	Symptomatic	EPD used	Nr of MAE's	MAE (%)
Micari [16]	2010	198	198	68%	83.2	39%	100%	5	2.5%
Grant [17]	2010	418	418	63%	83.2	32%	79%	14	3.3%
Bacharach [18]	2010	235	78	72%	83.5	20%	99%	7	9.0%
Usman* [19]	2009	826	826	NR	82.2	65%	100%	84	9.9%
Linfante [20]	2009	178	24	67%	82.4	71%	100%	2	8.4%
Cremonesi [21]	2009	1.523	237	72%	NR	24%	88%	5	2.1%
Jackson [22]	2008	215	35	53%	NR	41%	92%	4	11.4%
Henry [23]	2008	930	121	72%	82.0	64%	95%	2	1.6%
Velez [60]	2008	816	126	56%	82.9	40%	50%	3	2.7%
Zahn** [24]	2007	2.878	321	65%	82.5	61%	68%	18	5.5%
Lam [25]	2007	135	37	65%	85.0	100%	99%	4	10.8%
Villalobos [26]	2006	75	75	55%	83.1	56%	54%	18	24.0%
Stanziale [27]	2006	382	87	83%	83.0	18%	62%	8	9.2%
Setacci [28]	2006	1.222	144	75%	82.0	65%	92%	5	3.5%
Longo [29]	2005	158	29	74%	82.3	17%	89%	1	3.4%
Hobson [30]	2004	749	99	64%	NR	30%	100%	12	12.1%
Roubin [31]	2001	604	66	67%	NR	52%	100%	11	16.0%
Shawl [32]	2000	170	42	59%	NR	61%	0%	1	2.9%
Total		11.712	2.963	67%	82.9	49%	86%	203	6.9%

* Meta-analysis of CAS vs. CEA in octogenarians

** All non-fatal strokes and death

**MAE:** Major Adverse Events (disabling stroke, myocardial infarction or death)

**Table 2:** Carotid Endarterectomy (CEA) Study Collection and 30-day MAE Rates.

First author	Year	Total CEA	Octogenarians, N	Male	Mean age, y	Symptomatic	Nr of MAE's	MAE (%)
Lichtman [33]	2010	140.376	140.376	NR	83.0	NR	3.088	2.2%
Usman* [19]	2009	7.017	7.017	NR	82.7	54%	316	4.5%
Halm [34]	2009	9.308	2.198	NR	NR	NR	106	4.8%
Bremner [35]	2008	195	105	62%	83.7	43%	6	5.7%
Ballotta [36]	2006	1.260	112	62%	84.2	66%	1	0.9%
Teso [37]	2005	14.679	2.379	54%	NR	11%	100	4.2%
Pulli [38]	2005	1.883	149	70%	NR	NR	1	0.6%
Miller [39]	2005	2.217	360	NR	83.6	59%	15	4.2%
Lau [40]	2005	286	33	100%	82.0	51%	3	8.0%
Grego [41]	2005	1.733	125	66%	NR	50%	1	0.8%
Durward [42]	2005	1.800	26	NR	91.3	81%	0	0.0%
Varghese [43]	2004	359	33	61%	NR	76%	3	8.8%
Hingorani [44]	2004	565	299	51%	NR	43%	8	2.7%
Ballotta [45]	2004	1.150	92	52%	83.7	66%	0	0.0%
Witz [46]	2003	360	47	66%	82.0	51%	4	8.3%
Rockman [47]	2003	698	161	52%	NR	46%	4	2.5%
Pruner [48]	2003	3.430	269	62%	82.9	83%	8	3.1%
Norman [49]	2003	2.023	151	67%	NR	NR	4	2.6%
Salameh [50]	2002	293	42	NR	NR	NR	2	4.8%
Ozsvath [52]	2002	3.932	125	45%	83.0	50%	3	2.4%
Metz [53]	2002	32	32	50%	82.0	100%	1	3.2%
Cartier [54]	2002	475	65	51%	82.6	76%	2	2.8%
Saha [51]	2002	101	101	NR	86.5	71%	3	3.0%
Ommer [55]	2001	2.262	70	63%	82.9	74%	3	4.2%
Lepore [56]	2001	366	42	63%	82.8	40%	1	2.4%
Ting [57]	2000	656	57	58%	82.0	86%	4	6.8%
Schneider [58]	2000	582	88	61%	83.2	75%	1	1.1%
Maxwell [59]	2000	2.398	187	47%	83.0	65%	8	4.1%
Total incl. Lichtman *et al*		200.436	154.741	NR	83.5	47%	3.695	2.4%
Total excl. Lichtman *et al*		60.060	14.365	NR	83.5	NR	606	4.2%

* 30-day Mortality rates, stroke not analysed

** Meta-analysis of CAS vs. CEA in octogenarians **MAE:** Major Adverse Events (disabling stroke, myocardial infarction or death)
